# New tools and expanded data analysis capabilities at the protein structure prediction center

**DOI:** 10.1002/prot.21653

**Published:** 2007-08-17

**Authors:** Andriy Kryshtafovych, Andreas Prlic, Zinoviy Dmytriv, Pawel Daniluk, Maciej Milostan, Volker Eyrich, Tim Hubbard, Krzysztof Fidelis

**Affiliations:** Genome Center, University of CaliforniaDavis, California 95616; The Sanger Center, HinxtonCambs CB10 1SA, United Kingdom; Institute of Computing Science, Poznan University of TechnologyPoland; CUBIC, Department of Biochemistry and Molecular Biophysics, Columbia UniversityNew York, New York 10032

**Keywords:** CASP infrastructure, protein structure prediction, evaluation methods, SPICE

## Abstract

We outline the main tasks performed by the Protein Structure Prediction Center in support of the CASP7 experiment and provide a brief review of the major measures used in the automatic evaluation of predictions. We describe in more detail the software developed to facilitate analysis of modeling success over and beyond the available templates and the adopted Java-based tool enabling visualization of multiple structural superpositions between target and several models/templates. We also give an overview of the CASP infrastructure provided by the Center and discuss the organization of the results web pages available through http://predictioncenter.org

## INTRODUCTION

In CASP7, we received over 63,000 predictions in six prediction categories, including over 52,000 tertiary structure predictions. This constitutes ∼50% more predictions than in CASP6, and in terms of size of data, 30% more structures than presently held at the PDB (∼40,000). To analyze these predictions, a robust automated system for prediction processing, evaluation, and visualization of results is necessary. Building on the relational database system implemented for CASP6, and expecting another increase in the dataflow volume, we have improved the reliability of data processing. However, for CASP7 our primary emphasis was to make the system more transparent and easy to use. We have also broadened the results analysis toolkit. This article aims at outlining the automatic evaluation process and making easier navigating through the material available at the Prediction Center's website, and pays particular attention to changes introduced since CASP6.

## PROTEIN STRUCTURE PREDICTION CENTER IN CASP7

In the year following CASP6, the Prediction Center has moved from the Lawrence Livermore National Laboratory in Livermore, California to the University of California at Davis. However, the role played by the Center remained essentially unchanged:

- providing information about the CASP experiment;
- registration of participants;
- solicitation and selection of prediction targets for the experiment (i.e., soon to be solved protein structures);
- verification of the target data; release of targets for prediction;
- submission of targets to prediction servers;
- releasing of targets to human expert predictors;
- acceptance of protein models from servers and human-expert prediction groups; verification of prediction formats and compliance with submission/prediction correction deadlines;
- releasing server-generated models to expert predictors;
- monitoring of the public release of target coordinates, with particular emphasis on instances compromising the blind prediction principle of the CASP experiment;
- conducting the model refinement experiment;
- developing prediction evaluation methods;
- executing automatic evaluation of predictions;
- analyzing evaluation results;
- providing assistance to CASP assessors;
- corresponding with predictors and observers;
- publishing meeting materials;
- organizing the meeting at Asilomar.

### Registration of participants

CASP7 registration was open from early April until the end of August 2006, via the new Prediction Center website (http://predictioncenter.org). Registration rules were the same as in CASP6.^1^ Totally, 253 predictor groups representing 25 countries registered and submitted predictions. Approximately, the same number of human expert groups participated in CASP7 as in CASP6 (160 and 165, respectively), while server participation increased by about 50% (93 in CASP7 vs. 63 in CASP6).

### Prediction targets

Over 150 sequences were received from X-ray crystallographers and NMR spectroscopists during the course of the experiment. All accepted sequences were prescreened and 104 targets were selected by the organizers. We are grateful to all target contributors, especially to the four structural genomic centers (JCSG, MCSG, NESG, and SGC) which provided the majority of CASP7 targets by submitting 20+ sequences each (see http://predictioncenter.org/casp7/targets/forms/casp7-tar.html for the complete list of people/institutions contributing). We also owe our thanks to the Protein Data Bank for putting in place a mechanism for keeping some of the deposited structures on hold, with the aim of making them available as targets for CASP7.

The selected sequences were released for prediction in sets of maximum three targets per day (and 700 residues total), usually 4 days per week. Our automatic tracking system of released structures advised cancelling four targets due to their early release. Assessors additionally canceled five targets as impossible (no structure) or unsuitable (low quality or extended disorder regions) for evaluation. This way, 95 targets were left for assessment in CASP7.

In CASP7, we have finally reached the long-standing goal of 100 prediction targets. However, the increase in the number of targets resulted in a mixed reaction from the prediction community. According to our post-CASP polling, opinions roughly split in half. While many predictors, most probably representing server groups, were happy with 100 or more targets, there were also many who felt that there was not enough time for human input and that it was difficult to achieve good results for 100 targets in 3 months. As a compromise, at the Predictor's Meeting at Asilomar, it was decided that a mixed approach should be used in CASP8, that is, the organizers should release as many targets as possible for the server groups, and select a subset of these (50–60 targets) for the human-expert predictors.

### Accepting predictions

Prediction windows in CASP7 were in general the same as in CASP6 for servers (48 h) and shorter for human-expert groups (∼3 weeks). These limits were implemented to adhere more closely to the target structure release timelines adopted by crystallographers and to fit within the window designated by the “4-week CASP hold” agreement with the PDB. In the end, this approach helped to minimize information leaks and subsequent target cancellations (only 4 in CASP7 vs. 11 in CASP6). However, to allow assessment of methods requiring longer computation times, we have extended some target deadlines, mainly for the most difficult targets. In such cases, we encouraged predictors to submit their models by the 3-week “soft” deadline and possibly to resubmit later, but before the hard deadline. Thus, in situations when information leaks occurred after the first but before the second deadline, the evaluation could be limited to models submitted within the 3-week time window. This rule was enforced in CASP7 only once (Target T0295).

All predictions were collected, checked for format consistency, and stored in the relational database at the UC Davis Prediction Center. We accepted predictions in six categories (seven formats): protein tertiary structure (3D, comprising two different formats—TS, tertiary structure and AL, alignment to a PDB structure), residue–residue contacts (RR), disordered regions (DR), domain boundaries (DP), function predictions (FN), and model quality assessments (QA)—see http://predictioncenter.org/casp7/doc/casp7-format.html for all format details. The latter category was introduced in CASP7 whereas all others carry over from previous CASPs.

#### Tertiary structure predictions

There were several changes in the acceptance procedure and formats. We introduced a filter rejecting outright any human-expert prediction compromised by a severely unrealistic geometry. The criteria for rejection were as follows: more than 5% of C_α_s taking part in severe clashes (<1.9 Å) or more than 25% of C_α_s taking part in moderate clashes (<3.5 Å). If a prediction contained at least one severe clash (but less than 5%) or more than 10% of moderate clashes (but less than 25%), or it contained more than four chain breaks (C_α_s adjacent in sequence but separated by more than 4.5 Å in space), a warning was issued. In cases like that, predictions were accepted but annotated, leading to additional scrutiny by the assessors. Missing loops or other deletions were not considered as excessive fragmentation. Server predictions were not rejected based on these criteria but instead they were flagged.

#### Function predictions

Format for the predictions of function was changed, the main emphasis being placed on predicting the EC numbers rather than GO classifications. It was decided that in CASP8 the accent should be shifted once again, by asking predictors to focus primarily on protein binding sites.

#### Prediction of model quality

For a new prediction category, model quality assessments attracted considerable attention from CASP participants. Since CASP2, predictors had an opportunity to submit estimates of the per residue reliability of their own predictions, using the PDB's B-factor field in the CASP TS format. In addition to that, in CASP7, predictors were asked to estimate the overall and local correctness of models submitted by others. During CASP7, the models we had been receiving from the participating servers were being released through our web site on regular basis, following the server prediction time window. Predictors were asked to return the overall reliability score (between 0 and 1) and the per-residue error estimation in Angstroms for this collection of server models. The deadline for accuracy predictions was the regular deadline for each target (typically 3 weeks).

#### Prediction of quaternary structure

In CASP7, 13 targets were specified as possible oligomeric structures. In these cases, predictors could submit multichain predictions. We have provided a separate format for these predictions and submission that followed the general rules for monomeric predictions.

#### Model refinement

Eight targets from among the 95 in the regular CASP7 were selected for model refinement. The criteria were small target size, prompt availability and high quality of the experimental structure, availability of good models, and the ability to extend the prediction deadline beyond the typical 3 weeks. After the regular prediction for a particular target was completed, a single model submitted within the regular prediction time window was selected and released for refinement. Additional 3 weeks were granted to perform these calculations. Twenty six groups participated in the experiment submitting 447 predictions.

### Servers in CASP7

Compared with CASP6, in CASP7 we have counted more servers, more server predictions, and more prediction categories, in which servers participated. Overall, 93 servers participated in the experiment, including 68 in the 3D category, 14 in DP, 8 in RR, 8 in DR, and 6 in FN. In total, servers submitted

- 30648 predictions including 26647 3D, 1617 DP, 794 RR, 811 DR, and 779 FN predictions;
- 9905 models designated as first, including 6452 in 3D, 1399 in DP, 794 in RR, 811 in DR, and 449 in FN category.

These numbers represent an increase relative to CASP6 in all five prediction categories. Rules for accepting server predictions remained, in general, the same as in CASP6,^2^ although the system for handling the predictions was modified. In CASP6, we used an intermediate server at the Columbia University to send queries to participating servers, accept their responses, and forward the accepted models to the Prediction Center. In CASP7, we have sent target queries directly from the CASP distribution server in Davis, and accepted the models directly at the Prediction Center. This streamlined system eliminated possible problems with power failures, and so forth at the intermediate server. As before, all predictions were automatically checked for format compliance by the CASP verification software and error messages were automatically sent to server curators via email (while confirmation messages were suppressed in CASP7 complying with requests from predictors). We also improved prediction status pages enabling easier tracking of submitted predictions. Following closing of the server prediction window, we posted the server models at our website. These models could then be used by human-expert predictors. They were also used in the model quality assessment experiment (QA category).

### Structures used in evaluation

In cases where several structures were available, we have selected one with the best resolution. If the experimental structure appeared to be a multimer, it was analyzed in terms of chain similarity and the most typical chain and/or the one missing fewest residues was selected. NMR structures were checked for model agreement and variable zones were flagged. Structure's sequence and residue numbering were brought into agreement with the released sequences; chain IDs were stripped. Both processed and unprocessed target structures and all the available supplementary information (resolution, R-factor, space group, ligands, etc.) were provided to the assessors. Special infrastructure was enabled to allow the assessors to discuss target specifics, define prediction domains, and assign prediction categories. At this stage, we have also identified the best structural homologues for all the available target structures.

### Similarity measures and automatic evaluation of predictions

**Figure 1:**
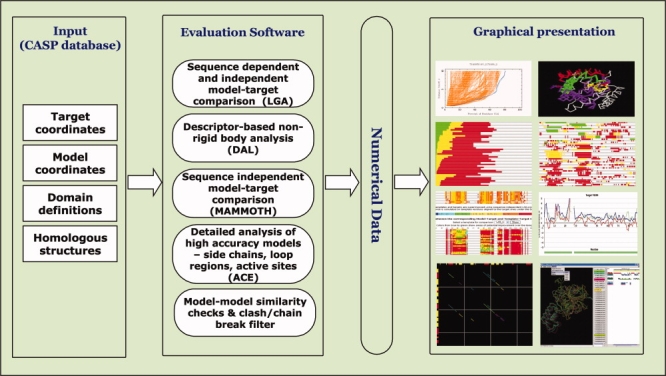
Schematic of the CASP7 prediction evaluation system. [Color figure can be viewed in the online issue, which is available at www.interscience.wiley.com.]

As soon as the target structures became available at the Center, we have performed the automatic evaluation of predictions (see Fig. 1). As in CASP6, we have used the structure comparison program LGA^3^ and the descriptor-based software descriptor-based alignment (DAL)^1^,^4^ to identify the best structural model-target superpositions in the rigid-body/nonrigid-body regimes, respectively. We have also used structure comparison program MAMMOTH^5^ to offer an alternative measure of prediction quality. Finally, we have used the ACE^6^ software to provide detailed evaluation of the template-based models.

LGA was run in both sequence-dependent and sequence-independent modes. In the sequence-dependent mode, the initial predefined correspondence between model and target residues is kept unchanged during the superposition process. Quality of prediction was measured with the GDT_TS score reporting the average percent of residues in a prediction that can be fitted to the target structure in four separate superpositions made with distance cutoffs of 1, 2, 4, and 8 Å, respectively. Another measure used in the template-based modeling (TBM) assessment was the GDT_HA. It is analogous to GDT_TS but compiled for a set of lower distance cutoffs (0.5, 1, 2, and 4 Å), providing a finer-grained estimate of quality for models built by homology. In the LGA sequence-independent mode, the preassigned correspondence between model and target residues is ignored and a new model-target alignment is generated in each iteration. Prediction quality was evaluated with the LGA_S^3^ score internal to the program, and the alignment accuracy score AL0 derived from the final superposition. AL0 reports percentage of model residues, for which the C_α_ atom falls within 3.8 Å of the corresponding C_α_ in the experimental structure, with no other experimental structure C_α_ nearer.

DAL^1^,^4^ is a structure comparison method designed to identify protein similarity using multiple frames of reference. Compared with rigid-body techniques, it provides a more comprehensive assessment of similarity, especially in cases where similar structure regions are oriented differently in the two compared proteins. In CASP7, the method was applied to all model-target comparisons. DAL_n scores are cumulative and correspond to the summation over all regions identified as similar in the two structures. DAL_0 corresponds to the case where superpositions are performed in the sequence-dependent mode, DAL_4—where a shift of up to four residues along the sequence is allowed, and DAL_I—where superpositions are fully sequence-independent.

Structure comparison program MAMMOTH^5^ was run to obtain *Z*-scores from sequence-independent structural alignments between models and targets. The algorithm is fast, allowing obtaining results for large scale structure comparison tasks.

Finally, we have used the ACE software package originally developed for CASP3 to provide detailed analyses of the high accuracy template-based models. In particular, information on the accuracy of side chain angles, core and loop regions, and ligand binding regions was obtained with this package.

To effectively manage the evaluation process, calculation tasks were semiautomatically distributed between processors in the cluster of CASP evaluation servers. Each process downloaded the necessary structures from the database and wrote the results into the central depository. From there, a set of Perl scripts parsed the results and uploaded the processed data into the database. These data were then used for generating dynamic tables and plots facilitating data analysis (easy sorting, selection, visualization, etc.).

### Organization of the website

The Protein Structure Prediction Center website provides general information about the prediction experiment as well as access to prediction targets, original predictions, evaluation results, and visualization. Data for all seven CASP experiments are available. For CASP7, three alternative views of the tertiary structure prediction data are made available: the target perspective view, the group perspective view, and the table browser. In addition, links to the results of the refinement and the quality assessment experiments are provided.

#### Target perspective view

**Figure 2:**
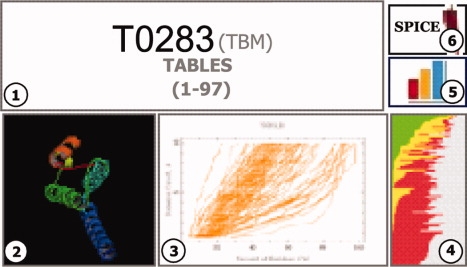
Information cell for target T0283. Clickable pictograms represent (1) tabulated numerical results, (2) 3D interactive representations of the target structures, (3) GDT plots, (4) alignment quality bar graphs, (5) comparison of models with template structures, and (6) SPICE-based interactive target/model/template visualization.

The target perspective view (http://www2.predictioncenter.org/casp/casp7/public/cgi-bin/results.cgi) is the default viewing mode and provides access to the results on the target-by-target basis. It can be reached from the main CASP7 web page or by selecting the Results Home link in the main menu bar located at the top of any results page. The main web page is designed so that miniature plots allow an at-a-glance comparison between all evaluated targets/domains. Results for each target are collected in “information cells” consisting of six clickable pictograms (see Fig. 2). Later on we discuss the results presented for each target, paying particular attention to the newly introduced value-added plots and the SPICE visualization tool.

1. The **tables** pictogram display the target/domain number, difficulty category (TBM for template-based modeling or FM for free modeling, and the range of target residues used in evaluation. Clicking on the pictogram allows reaching tabulated results for all predictions accepted on a given target. Tables may be sorted by each column (by clicking on the small black triangle next to the measure name) and may be expanded or contracted with the Full/Brief option. Model IDs reflect target, prediction group, and model (1–5) numbers. The results are grouped in four blocks comprising data from the LGA sequence-dependent and sequence-independent analyses, MAMMOTH sequence-independent, and DAL local structure analyses. LGA and ACE results text files, interactive renderings of LGA-based model-target sequence dependent and sequence-independent superpositions, and links to the GDT plots are provided through the A, D, I, and G links, respectively, in the General/Charts section. The links 2D, T, M, and <number_of_residues_in_the_substructure> in the DAL block, point, respectively, to the alignment maps(2D), the aligned substructures highlighted in target (T) and model (M) structures, and the model-target superpositions for each identified region of similarity (<number_of_residues_in_the_substructure>). Detailed descriptions of all measures can be reached via links associated with measure names. They are also discussed in more detail in the CASP6 evaluation paper.^1^
2. **3D interactive representations of target structures** may be viewed with visualization software (e.g., Rasmol^7^) installed on a local workstation. The evaluated areas are colored green. There are two reasons why some areas of the target may not be colored: (a) the target was split into domains and only the domain in question is highlighted, and (b) some residues were eliminated from evaluation by the assessors.
3. **GDT plots** provide prediction quality analysis by finding the largest subsets of residues in the model that can be fitted to the target in a series of rigid-body sequence dependent superpositions. The calculation is performed for cutoffs from 0.5 to 10.0 Å. Results are plotted as a line for each model separately. Models from several groups may be displayed simultaneously; and conversely, clicking on any line in the plot will identify the corresponding model and research group (dark blue indicates Model 1, cyan – all other models submitted by the group).
4. The **alignment summary** strip charts indicate percentages of correctly aligned residues in the model relative to target (green), those aligned with an error not larger than four residues (yellow), and five residues or more (red). The bars under the position-specific alignment tab show the distribution of the correctly and incorrectly aligned residues along the target protein sequence. Clicking on each bar produces a 3D interactive rendering of the superimposed model and target in the best rigid body sequence independent superposition. Further details and definitions are provided through links in the graphs.
5. The **value-added plots** were implemented to help identify model features that are not available from a single template. LGA sequence-independent protocol was used to superimpose best templates onto the target structure. The analysis page with the three main tabs, *selected templates/models*, *models strip charts*, and *templates strip charts* allows relating quality of every submitted model to the structural information available from the 10 best structural templates.
  The *selected templates/models* tab provides a line graph depicting distances between the aligned C_α_ residues in a model (blue) or template (green) and the corresponding experimental structure, and a strip chart representation of this graph. User can select a model and several templates to be displayed at once. The graphs also show the difference between the corresponding model–target and template–target C_α_–C_α_ distances (red line). The difference line can be displayed for only one template at a time. Red line negative values represent regions where a model has a better fit to the target than the selected template, that is, areas of potential improvement over the template. The percentage strip chart gives a summary of all the displayed results. Numerical results are also provided for each model/template in the selection table.
  *Models strip charts* show regions where model structure is closer to the target than available templates. These graphs can also help identify models that were built using several templates. The top plot shows C_α_–C_α_ distances calculated from sequence-independent superpositions of the target and best 10 structural templates. The bottom plot shows submitted models plotted versus the target sequence and compared with the selected template (from the “best 10” list) for that target (see Fig. 3). Clicking on the bar shows 3D Rasmol superposition of the target (thick line), model (thinner line), and the selected template (thinnest line). Coloring in the Rasmol presentation is as follows: regions where the corresponding C_α_ residues of the target and model are less than 2/4/8 Å apart are colored green/yellow/orange, respectively.

**Figure 3:**
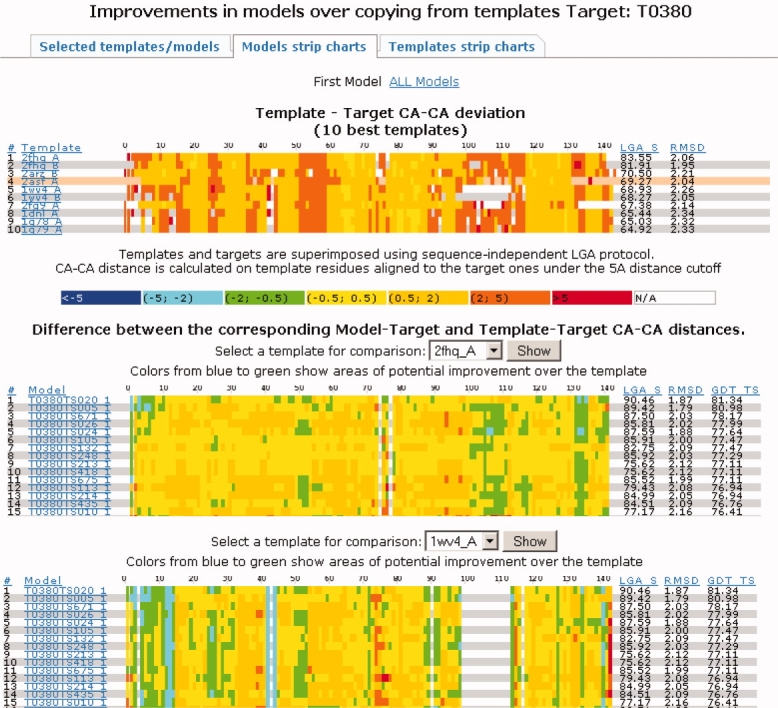
Strip charts displaying target–template-model proximity. Colors from blue to green show areas of potential model improvement over the template.
  *Templates strip charts* illustrate the availability of templates for each residue of the target sequence. Template–target C_α_–C_α_ deviation strip chart is shown at the top of the page to facilitate comparisons. In the bottom graph, for each residue, the height of a bar shows how many templates out of best 10 cover that particular residue in a structural alignment.
6. The **SPICE visualization tool** allows viewing of several model structures and/or corresponding target structures and templates in the same frame of reference. SPICE is a browser designed to annotate protein sequences and structures,^8^ based on the distributed annotation system (DAS).^9^ We have adapted SPICE for this CASP to allow visualizing multiple protein-structure alignments. This is particularly important when CASP targets, corresponding predictions, and the closest templates are analyzed simultaneously. As part of our evaluation, alignments between all these structures are computed. These data are available via DAS servers and can be viewed and compared in 3D using SPICE. The CASP target summary page provides links launching SPICE using the Java Web Start technology. There are three types of CASP DAS servers: (1) a structure-DAS server providing the coordinates for all the structures; (2) alignment-DAS servers providing information about which predictions have been made for a particular target and how to rotate and shift the structures so that they can be superimposed with the target. Three different alignment methods have been used for the evaluation of all the submissions. SPICE supports switching between the results of the algorithms. This is achieved by providing a separate alignment DAS server for each of the alignment methods; (3) feature-DAS servers providing information about how well particular regions of a prediction match the target structure.

The results are available for the full-length targets, as well as for the targets split into subdomains. This creates about 30 GB of flat-file data. In CASP7, for a typical target, 500–600 predictions were submitted (100+ predictors submitting up to five models). To provide fast access to these data, the DAS servers process and cache the evaluation data in a local database. This process takes about 1 h, but results in a much improved response time for the servers.

**Figure 4:**
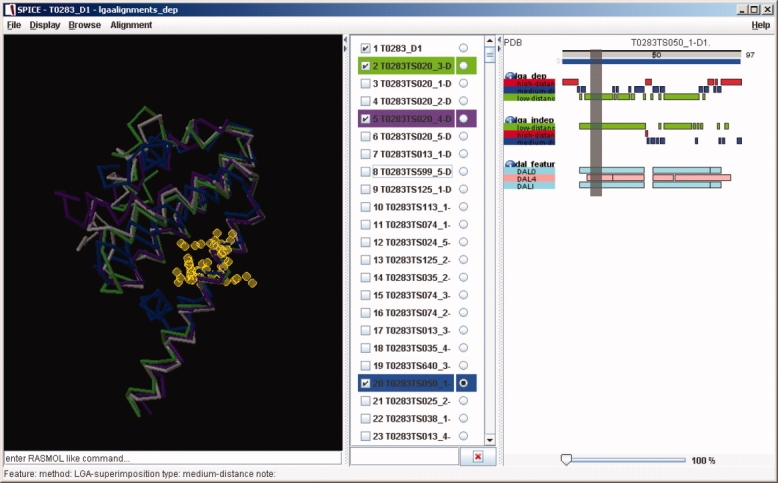
A screenshot of SPICE visualization server. The target/predictions/templates chosen in the middle panel are shown in the left-hand side structure display panel in a common frame of reference; the right-hand side panel displays the sequence of the currently active prediction and is interactively connected with the structure display panel: selecting sequence regions in the right-hand side panel highlights the corresponding regions in the protein structure.

The SPICE display consists of three sections (Fig. 4): (1) a 3D protein structure display, which is based on the Jmol library (http://jmol.sourceforge.net/) and allows to be interacted with by using RASMOL style scripting commands. (2) The middle display showing the current target chosen for display, as well as all available predictions. Multiple predictions can be selected simultaneously. Their structures are downloaded on demand and their superimposition shown in the structure display. (3) The feature display showing the sequence of the currently displayed prediction and the proximity of a particular region to the template according to each of the three alignment methods. Regions of close similarity are shown in green and large distances are in red.

#### Alternative views of the results

In addition to the target-perspective view, the CASP7 system incorporates two other views of the structure prediction results, the Groups view and the Table Browser. It also provides access to the refinement and quality assessment data. Users can switch between the five access modes using the menu bar at the top of any results page.

The Groups view allows assessing performance of a particular prediction group. It is possible to retrieve dynamically generated tables and graphical results over all targets predicted by that group. Results are shown in the context of all other submissions. In addition, GDT graph pages allow direct visual comparison of up to four groups.

The Table Browser view adds additional flexibility in generating custom comparisons of numerical results, where prediction groups, targets, and measures may be independently selected. The tables also provide links to graphical representations. It is possible to choose only server predictions for this type of analysis.

The refinement results access mode provides analyses performed on all eight CASP7 refinement targets. For each target, strip charts show improvements over the starting model. The refinement target (experimental structure) is superimposed with the refined models and the starting model using sequence-dependent LGA protocol with 4 Å distance cutoff. Colors in the bars are arranged from blue to red showing the accuracy of the C_α_–trace in the refined model relative to the starting model, that is, the differences between the C_α_–C_α_ distance in the two corresponding superpositions: refined_model – target, and starting_model – target.

The quality assessment results access mode provides analyses of the automatic evaluation of model quality predictions. Data on both the overall and residue-by-residue correlation of QA predictions with actual results are provided.
